# Design of Broadband Flat Optical Frequency Comb Based on Cascaded Sign-Alternated Dispersion Tellurite Microstructure Fiber

**DOI:** 10.3390/mi12101252

**Published:** 2021-10-15

**Authors:** Guocheng Huang, Meicheng Fu, Junli Qi, Jinghan Pan, Wenjun Yi, Xiujian Li

**Affiliations:** Department of Physics, College of Liberal Arts and Sciences, National University of Defense Technology, Changsha 410073, China; hgc@nudt.edu.cn (G.H.); fumeicheng10@nudt.edu.cn (M.F.); qijunli_r@nudt.edu.cn (J.Q.); panjinghan18@nudt.edu.cn (J.P.); yiwenjun@nudt.edu.cn (W.Y.)

**Keywords:** optical frequency comb, microstructure fiber, nonlinear optics, cascaded sign-alternated dispersion

## Abstract

We designed a tellurite microstructure fiber (TMF) and proposed a broadband optical frequency comb generation scheme that was based on electro-optical modulation and cascaded sign-alternated dispersion TMF (CSAD-TMF). In addition, the influence of different nonlinear effects, the ultrashort pulse evolution in the CSAD-TMF with the anomalous dispersion (AD) zones and the normal dispersion (ND) zones were analyzed based on the generalized nonlinear Schrodinger equations (GNLSE) modelling. According to the simulations, when the input seed comb had a repetition rate of 20 GHz and had an input pulse peak power of 30 W, the generation scheme could generate optical frequency combs with a 6 dB spectral bandwidth spanning over 170 nm centered at 1550 nm. Furthermore, the generated combs showed good coherence in performance over the whole 6 dB spectral bandwidth. The highly coherent optical frequency combs can be used as high-repetition-rate, multi-wavelength light sources for various integrated microwave photonics and ultrafast optical signal processing applications.

## 1. Introduction

The optical frequency comb (OFC) refers to a series of discrete, equally spaced frequency components, which covers a variety of applications such as optical frequency metrology, laser ranging, and astronomical spectrograph calibration [1,2,3]. Based on various applications, several schemes have been demonstrated to generate OFCs, including a mode-locked laser [4,5], an electro-optic frequency comb [6,7,8], and a Kerr comb [9,10]. For the unique advantages of high repetition rates, spectral flatness, robustness, and reconfiguration flexibility, the electro-optic frequency comb has received considerable attention as a valid tool for numerous RF photonic applications such as wavelength division multiplexing, optical arbitrary waveform generation, and all-optical signal processing [11,12,13,14]. The electro-optic frequency comb can be generated by various methods, including the use of a dual-drive Mach–Zehnder modulator (MZM) [15], cascading intensity modulators (IM), phase modulators (PM) [16], time-to-frequency mapping [17], and so on, in which the comb lines number and bandwidth coverage are limited by the modulator’s bandwidth and the performance of the RF source.

To promote comb bandwidth, highly nonlinear mediums have been applied for nonlinear broadening. By utilizing 150 m of highly nonlinear fiber (HNLF) with normal dispersion profile, a 10 GHz ultra-broadband comb (28 nm bandwidth within 3.5 dB power variation) generator was demonstrated [16]. In 2020, based on a precise parameter mixer setup, a sub-100 fs all-fiber electro-optic OFC which had a 6 dB spectral bandwidth spanning over 150 nm was demonstrated [8]. Recently, a non-coherent broad seed comb was generated by using a single MZM, then an OFC of 55 frequency lines with 3 dB flatness was obtained via four-wave mixing in 400 m of HNLF [18]. Another study demonstrated that, via sign-alternated dispersion, the stagnation of spectral broadening can be eliminated, and the required power can be significantly reduced [19]. However, most of the current schemes rely on silica highly nonlinear fiber (SHNLF) [6,15,20], in which the inherent nonlinear refractive index limits the improvement of the nonlinear coefficient (10~20 W^−1^km^−1^), and the lengthening of the SHNLF will reduce the threshold of SBS and cause the zero-dispersion wavelength to shift. Fortunately, a growing number of new materials with a higher nonlinear coefficient and broader transmission windows have been demonstrated recently [21,22,23]. Among them, tellurite microstructure fiber (TMF), which is based on 70TeO_2_-20BaF_2_-10Y_2_O_3_ glasses, has a high nonlinear refractive index, stable chemical and thermal properties, high damage threshold [24], and a large transparent band ranging from the visible band to the long-wave infrared (=5 μm) band. Furthermore, the nonlinearity coefficient of the TMF is at least one order of magnitude higher than that of the SHNLF [25]. All of the above unique properties of TMF reveal its great potential in supercontinuum and frequency comb generation [26,27].

In previous work [28], a flat-top optical frequency comb covering 1500–1600 nm was generated by using a 2 m long, near-zero dispersion fluoroantimonate microstructure fiber. However, the results show that the fiber dispersion was greatly affected by the inner air hole parameter, and that it was hard to keep near-zero dispersion. Herein, we proposed an efficient scheme based on a designed cascaded sign-alternated dispersion TMF (CSAD-TMF) with cascaded normal dispersion (ND) zones and anomalous dispersion (AD) zones to overcome this limitation.

**Figure 1:**
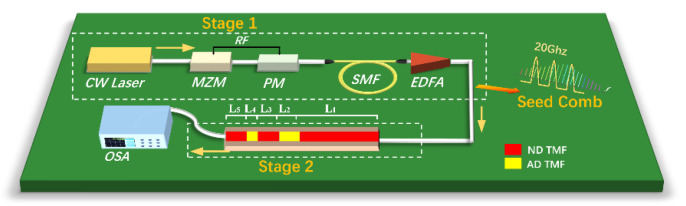
Schematic diagram of a broadband flat OFC generator based on CSAD-TMF.

As shown in Figure 1, the schematic diagram of a broadband flat OFC generator based on the CSAD-TMF can be divided into two stages: Stage 1 and Stage 2. In Stage 1, the seed frequency comb is generated by a dual-drive MZM and a PM driven by a 20 GHz RF signal with equal phase. Here, the half-wave voltage at the DC bias voltage port and the RF drive port of the dual-drive MZM are set to 5 V and 1.6 V, respectively. The output optical signal has a positive chirp after passing through the PM, then a 290 m single-mode fiber is set for chirp elimination to perform ultra-short pulses simultaneously. The high-power erbium-doped fiber amplifier (EDFA) lifts the pulse peak power up to 30 W. In Stage 2, the generated seed comb passes through a sign-alternated dispersion TMFs with five cascaded ND and AD fiber segments with different designed lengths, which broaden the output spectrum through nonlinear effects such as four-wave mixing and self-phase modulation.

## 2. Generation of Electro-Optic Seed Comb

**Figure 2:**
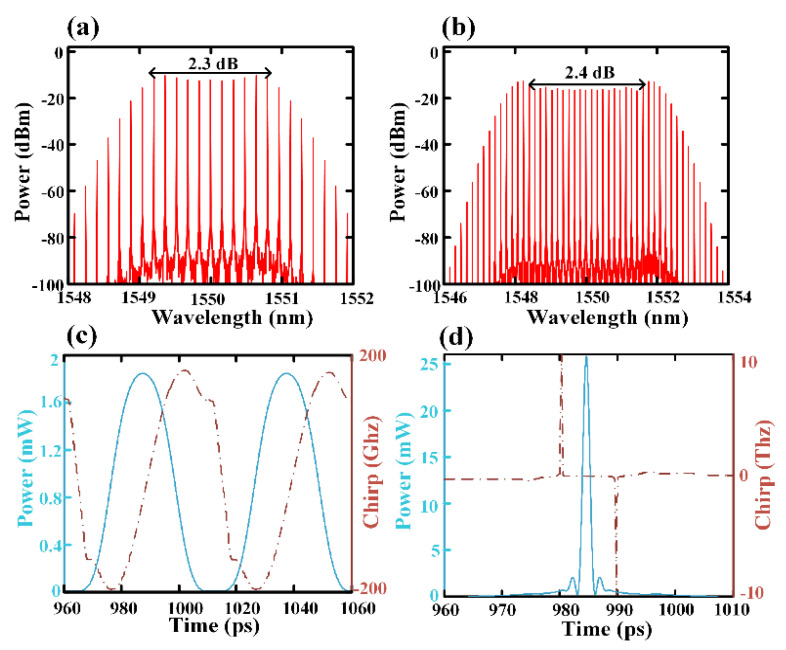
(**a**) The spectrum of OFC generated by a single MZM; (**b**) the spectrum of OFC generated by cascaded dual-drive MZM and phase modulator; (**c**) the time domain of the pulse after phase modulator; (**d**) the time domain and chirp of the pulse after the compression of single-mode fiber.

The center wavelength of the CW laser in Stage 1 was 1550 nm with linewidth of 20 kHz, and the output power was 10 dBm. The output optical signal of the dual-drive MZM can be expressed as:

(1) $$E_{\mathrm{mzm}}=\frac{1}{2}E_{in}\sum_{k=-\infty }^{+\infty }\left[J_{k}(\alpha_{1})e^{i(\omega t-\Delta \phi )}+J_{k}(\alpha_{2})e^{i(\omega t+\Delta \phi )}\right],$$

where $E_{in}$ is the amplitude of the input RF signal with frequency of ω, $J_{k}$ is the k-order Bessel function, and $\alpha_{i}=\pi \frac{V_{i}}{V_{\pi ,RF}}(i=1,2)$ represents the modulation index of two input RF signals. $\Delta \phi =\pi \frac{\Delta V_{DC}}{V_{\pi ,DC}}$ represents the phase difference introduced by two DC bias ports, in which the DC bias voltage difference $\Delta V_{DC}$ is set to 3 V for the following analysis. According to the measurements, the driving voltages $V_{1}$ and $V_{2}$ were set to 6 V and 4.65 V for the simulation, respectively. Under the above conditions, a flat OFC with 11 comb lines with 2.3 dB flatness can be observed in Figure 2a. After the phase modulator, the signal can be expressed as:

(2) $$E_{out}=E_{mzm}\sum_{k=-\infty }^{+\infty }J_{k}(\alpha_{3})e^{ikwt},$$

where $\alpha_{3}=\pi \frac{V_{3}}{V_{\pi ,PM}}$ is the phase deviation of the phase modulator, and $V_{\pi ,PM}$ is the corresponding half-wave voltage. When the phase deviation was set to 5π, 21 comb lines with 2.4 dB flatness was obtained, as shown in Figure 2b,c. Then, after the 290 m single-mode fiber that followed the phase modulator, which was used for chirp elimination, the compressed shortest pulse was obtained, wherein FWHM was ~1 ps, as shown in Figure 2d, in which the dotted line gives the chirp of the pulse. The chirp of the main pulse was completely eliminated after compression, but there were still some low power sidelobes in the time domain, which may have affected the spectral flatness of the OFC after Stage 2.

## 3. Design and Optimization of CSAD-TMF

**Figure 3:**
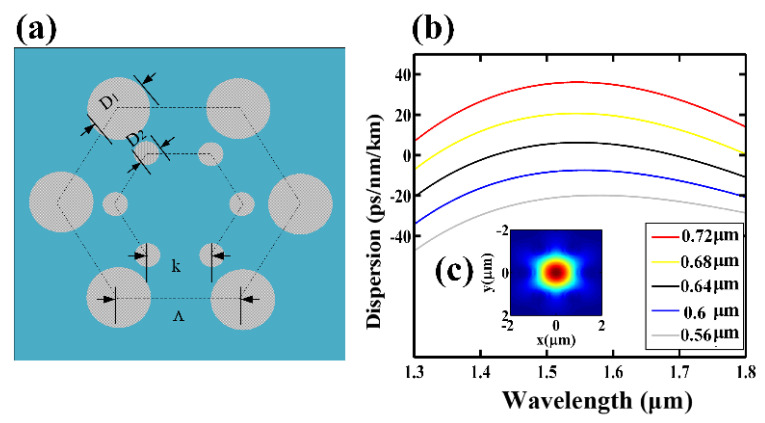
(**a**) Cross-section of the TMF; (**b**) the dispersion of the TMF when D_2_ changes from 0.56 µm to 0.72 µm for k = 1.27 µm; (**c**) the field profiles of the fundamental modes in TMF.

To achieve a flat OFC, the sign-alternated dispersion TMF in Stage 2 was finely designed. As shown in Figure 3a, the TMF core is surrounded by two layers of regular hexagonal air holes. The diameter of the outer air holes is D_1_ and the hole spacing is Λ, while the diameter of the inner air holes is D_2_ and the hole spacing is k, which can be finely tuned to adjust the dispersion of the TMF effectively.

To achieve a zero dispersion wavelength of 1550 nm, D_1_ was set to 3.8 µm and Λ was set to 4 µm according to the dispersion regulation in [28]. Furthermore, the inner holes can be finely adjusted for obtaining a flatter normal dispersion and anomalous dispersion profile over the C-band. The inner hole spacing k was optimally set to 1.27 µm. The inner holes diameter D_2_ was further finely adjusted. It was found that when D_2_ was increased from 0.56 µm to 0.72 µm, the dispersion at 1550 nm would transform from the ND to the AD with nearly the same dispersion slope, as shown in Figure 3b. Figure 3c shows the field distribution of the fundamental transverse electric mode (TE) at the cross section of the TMF for D_2_ = 0.6 µm. Most of the energy was concentrated in the core of the TMF with the effective mode field area A_eff_ = 2.11 µm^2^. The calculated nonlinear coefficient is given by $\gamma =\frac{2\pi n_{2}}{\lambda A_{eff}}$, where $n_{2}$ is the nonlinear refractive index and λ is the working wavelength. According to the above analysis, we selected D_2_ = 0.6 µm for the ND zone and D_2_ = 0.72 µm for the AD zone. The main simulation parameters, including the second order dispersion, the third order dispersion, effective field areas, nonlinear coefficients of the TE modes, and the transmission loss, are listed in Table 1.

## 4. Numerical Simulations

**Figure 4:**
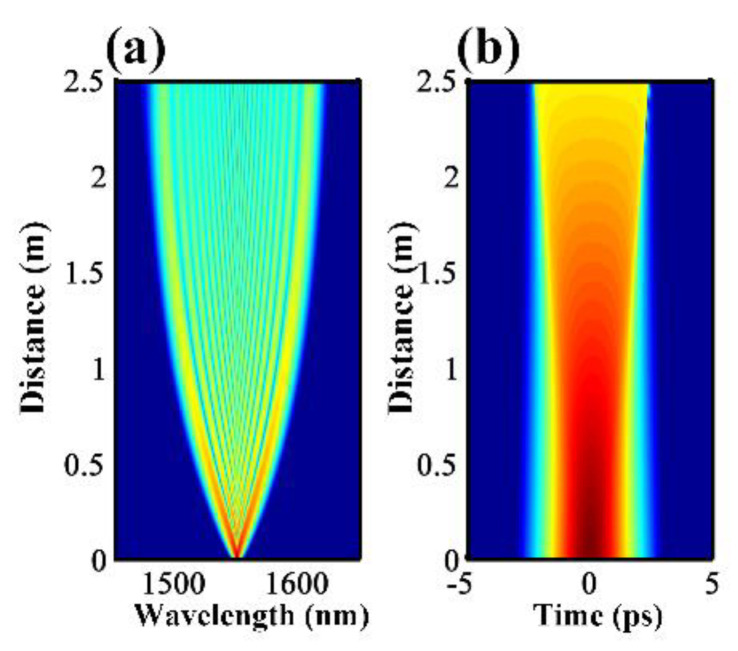
(**a**,**b**) Evolution of pulses and spectrum in a 2.5 m ND TMF.

To analyze the pulse evolution in the CSAD-TMF, numerical simulations were performed by solving the generalized nonlinear Schrodinger equations (GNLSE). Firstly, the transmission characteristics of an unchirped hyperbolic secant pulse in the designed TMF were analyzed. The FWHM of the input pulse was 1 ps, its center wavelength was 1550 nm and peak power was 30 W. In the simulation, the dispersion above the fourth order was ignored, and the fourth order Runge-Kutta algorithm was used to reduce the iterative error. Figure 4a,b show the evolution of the single pulse transmitting through a 2.5 m ND TMF. When the compressed pulse passes through the ND zone, owing to the SPM and optical wave breaking (OWB) [29,30], the pulse will continuously expand in the time domain and the frequency domain with increasing transmission distance up to a certain value. This leads to the formation of a flat frequency domain envelope, as shown in Figure 4a. When the transmission length is less than 2 m, the spectrum of the pulse continues to be widened with the increase of the transmission length, and the broadening in the blue shift and the red shift directions are roughly symmetrical. When the transmission distance is between 2 m and 2.5 m, the spectrum broadening has basically ended.

In the ND zone, the maximum spectrum width of the generated OFC and the splitting distance can be estimated by the following empirical formula:

(3) $${\left|f_{SPM}\left(L_{OWB}\right)-f_{0}\right|}_{max}\infty {\left(\gamma P_{0}/|\beta_{2}|\right)}^{1/2},$$

(4) $$L_{OWB}\infty T_{0}\sqrt{1/\gamma P_{0}|\beta_{2}|},$$

where $f_{SPM}$ is the maximum value of spectrum broadening contributed by the SPM, and $f_{0}$ is the center frequency of the pulse. It can be observed from Equation (3) that, in the ND region, the spectrum broadening width depends on the nonlinear coefficient, the GVD value, the pulse width, the peak power of the input pulse, and the transmission distance required for the spectrum envelope flattening by the OWB.

Theoretically, larger spectrum broadening can be obtained by appropriately increasing the input power and reducing the pulse width. However, it is difficult to obtain high-power short pulses and the tolerance of the material itself is also limited. High energy consumption is also not conducive to system integration. Here, we use CSAD-TMF to optimize the pulse evolution and the spectrum broadening.

**Figure 5:**
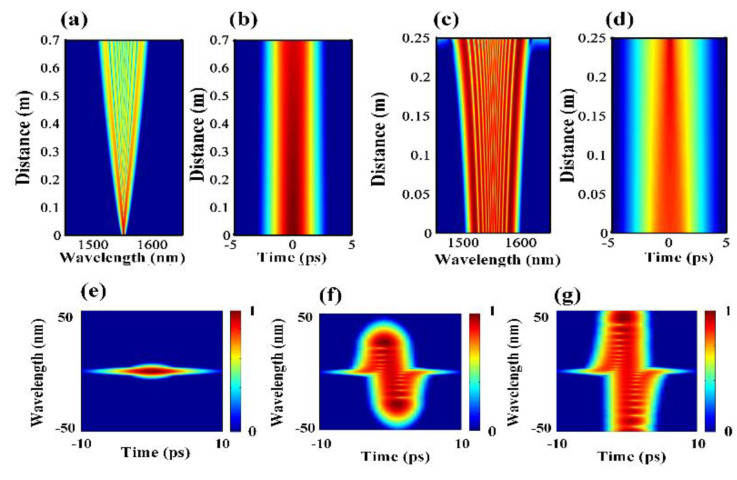
(**a**,**b**) Evolution of the pulses and the spectrum in 0.7 m ND TMF; (**c**,**d**) evolution of the pulses and the spectrum in 0.2 m TMF; (**e**) the initial pulse trace; (**f**) the pulse trace after the ND zone; (**g**) the pulse trace after the AD zone.

The evolution results of the same pulse in the CSAD-TMF are given in Figure 5. The length of the ND TMF and the AD TMF were determined as 0.7 m and 0.2 m, respectively, after optimization. The ND zone and AD zone can be connected by a tapering process [31], and the connection loss is ignored. In the 0.7 m ND zone, the pulse was broadened in both the time domain and the frequency domain, and the peak power of the pulse decreased simultaneously, as shown in Figure 5a,b. However, the flat top pulse envelope did not appear because of the short transmission distance. Then, the AD TMF was introduced to compress the pulse width and promote the peak power. In the AD zone, soliton compression occurs due to anomalous dispersion and SPM. The soliton order *N* is defined as follows:

(5) $$N=\sqrt[{2}]{\frac{L_{D}}{L_{NL}}}=\sqrt[{2}]{\frac{YP_{0}T_{0}^{2}}{\left|\beta_{2}\right|}},$$

where $L_{D}=T_{0}^{2}/|\beta_{2}|$ is the dispersion length, which depends on the input pulse width $T_{0}$, and the group velocity dispersion value of $\beta_{2}$. $L_{NL}=1/\gamma P_{0}$ is the nonlinear length and is decided by the nonlinear coefficient γ and the peak input power $P_{0}$.

For *N* = 1, i.e., the fundamental soliton, the group velocity dispersion (GVD) effect and the self-phase modulation (SPM) effect were balanced, where the shape and the spectrum of the pulse remained unchanged. For *N* ≥ 2, i.e., the higher-order soliton, the SPM played the major role at the beginning, which caused a red shift on the leading edge and a blue shift on the trailing edge of the pulse. In the AD zone, the transmission speed difference between the red spectral components and the blue spectral components resulted in the compression of the pulse and the broadening of the spectrum, as shown in Figure 5c,d. However, when broadening of the spectrum happens, the dispersion will become dominant and even soliton splitting will occur. When the transmission distance exceeds 0.25 m, soliton fission occurs and the flatness of the output comb is destroyed. Therefore, the length of the AD zone was set to 0.2 m.

The spectrograms of the output pulse after the ND zone and the AD zone were calculated using the XFROG technique, as shown in Figure 5e,g. The spectrogram of the initial pulse is given in Figure 5e. In the ND zone, the red spectral components travelled faster and became the leading edge of the pulse, which combined with the center wavelength component to produce new frequency components. A similar phenomenon can also be observed at the trailing edge of the pulse. As a result, some of the energy at the center wavelength of the pulse shifted to both ends of the spectrum continuously, which can be observed in Figure 5f.

In the AD zone, the pulse evolution is mainly determined by the soliton compression, in which the compression of the pulse leads to energy concentration and broadening of the spectrum. After the transmission in the CSAD-TMF (0.7 m ND TMF and 0.2 m AD TMF), the spectrum was broadened by more than 100 nm, as shown in Figure 5c,g. In future research, more sign-alternated subintervals will be added for further optimization.

**Figure 6:**
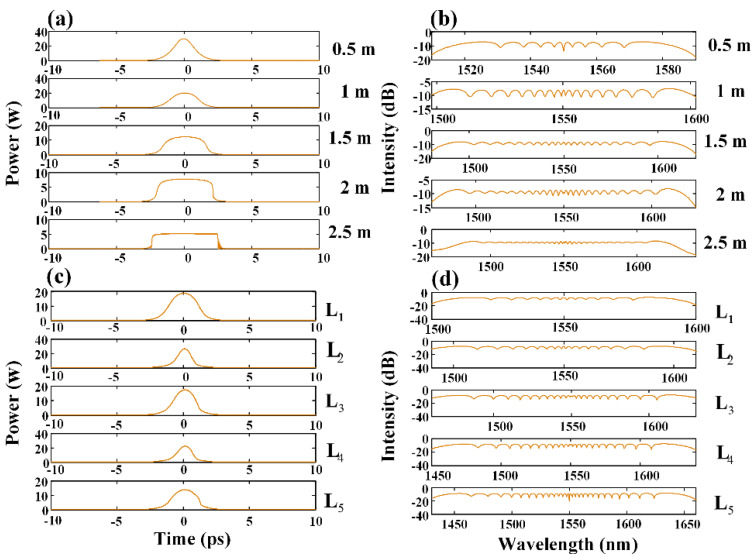
(**a**,**b**) Evolution of pulses at different positions of 2.5 m ND TMF; (**c**,**d**) Evolution of single pulse after different ND and AD zones of optimized CSAD-TMF.

The evolution of a single pulse in the ND TMF and the optimized CSAD-TMF is discussed below. Figure 6a,b show the pulse evolutions when the length of the ND TMF is 0.5 m, 1 m, 1.5 m, 2 m, and 2.5 m, respectively. In the time domain, the linear positive chirp provided by the normal dispersion broadened and reshaped the pulse into a rectangular pulse with an approximate flat top, while the leading anti-trailing edges became steeper. The corresponding spectrum was widened to cover 1500–1600 nm, and then the flatness was gradually improved when the transmission length exceeded 2 m. Finally, a flat optical frequency comb in the range of 1500–1600 nm was obtained.

For the CSAD-TMF, the length of each segment is *L*_1_ = 0.7 m (ND), *L*_2_ = 0.2 m (AD), $L_{3}=0.3\;m\;$(ND), $L_{4}=0.05\;m$, and $L_{5}=0.3\;m$, respectively, as shown in Figure 1. In Figure 6c,d, *L_n_* (*n* = 1,2,3,4,5) in the figure represents the output result when passing the nth segment in the CSAD-TMF. According to the pulse evolutions and spectrum shown in Figure 6c,d, the AD zones $L_{2}$ and *L*_4_ compressed the pulse, but the corresponding spectrum broadening was not obvious. After the ND zone$\;L_{1}$, the spectrum covered 1520–1580 nm, while after the ND zone *L*_3_, the spectrum was broadened and covered 1480–1610 nm, which was 70 nm wider than the spectrum after the ND zone$\;L_{1}$. For the ND zone, $L_{5}$ the spectrum broadening was only about 10 nm more than the ND zone $L_{3}$, but the flatness was significantly improved. Finally, a flat optical frequency comb with a spectral range of 1460–1650 nm was obtained.

Besides the spectral width and flatness, the coherence is another significant criterion to evaluate the performance of OFC, especially for applications such as super-high-capacity optical transmission based on WDM and TDM. Noise effects can be rigorously analyzed through the inclusion of stochastic variables to characterize quantum-limited shot noise [32]. Theoretically, the coherence of the OFC can be calculated by:

(6) $$|g_{12}(\lambda )|=\left|\frac{\langle \left|{E1}^{*}(\lambda )\right|E_{2}(\lambda )\rangle }{{[\langle {\left|E_{1}(\lambda )\right|}^{2}\rangle \langle {\left|E_{2}(\lambda )\right|}^{2}\rangle ]}^{1/2}}\right|,$$

where the angular brackets denote an ensemble average over independently generated pairs of OFC spectra and $\left(E_{1}{(\lambda ),E}_{2}\left(\lambda \right)\right)$. $|g_{12}(\lambda )|=1$ represents the full coherence of the OFC.

**Figure 7:**
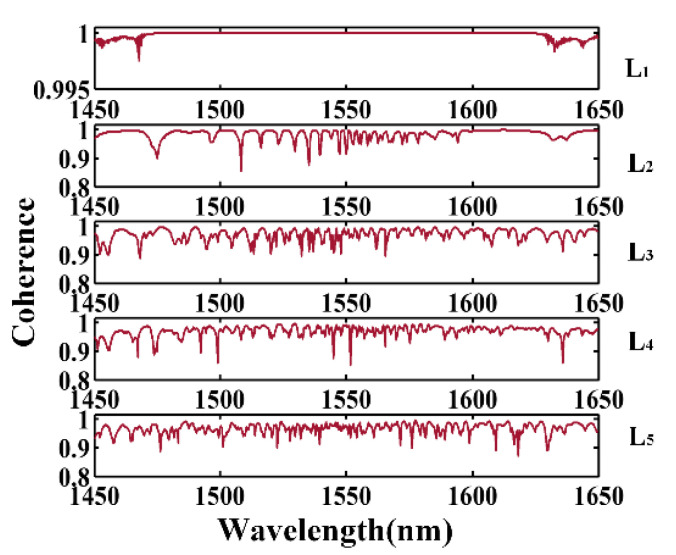
The coherence of the generated OFC from 1450 nm to 1650 nm after different ND and AD zones of optimized CSAD-TMF.

Figure 7 shows the calculation results of OFC coherence at the different transmission stages of CSAD-TMF. In the output of the AD zone $L_{1}$, the coherence in the range of 1450–1650 nm was close to 1, as shown in Figure 7. At the end of AD zone $L_{2\;}$, the coherence degraded, even dropping to below 0.9 at 1510 nm and 1535 nm, as a result of the modulation instability in the AD zone, which amplified both the coherent frequency component and the random noise. The AD zone TMF will degenerate the coherence of the OFC, while the ND zone TMF will make the distribution of coherence over the whole spectral band more consistent. It can be seen that in the output of the CSAD-TMF, the OFC coherence remained mostly above 0.9 in the range of 1450–1650 nm, which indicated that the OFC produced by this scheme had good coherence, and the phase relationship between different frequency components was stable.

**Figure 8:**
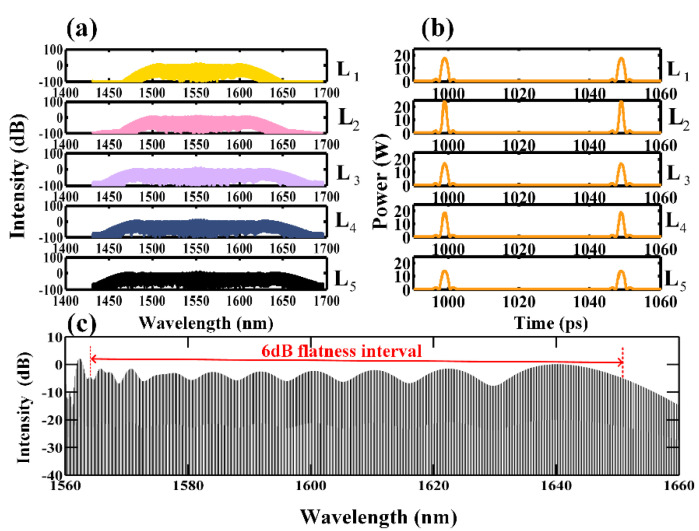
(**a**) The output spectra at different stages of CSAD-TMF; (**b**) the evolution of pulse profiles at different stages of CSAD-TMF; (**c**) the enlarged image of the final output spectrum over 1560–1660 nm.

The evolution of a seed electro-optic OFC with a repetition rate of 20 GHz in the designed CSAD-TMF is demonstrated in Figure 8. After the transmission distance $L_{1\;}$, spectrum broadening occurred between 1550 and 1600 nm because of SPM, OWB, and a cascaded four-wave mixing (FWM) effect in the ND zone, while the peak power of the pulse was attenuated from 30 W to approximately 18 W. After the transmission distance $L_{2\;}$ in the AD zone, the peak power of the pulse increased to approximately 25 W while the pulse width compressed. As a result of the cascaded pulse compression, dispersion compensation, and broadening in the AD zones and in the ND zones, at the output of the ND zone $L_{5}$, a flat top optical frequency comb covering 1460–1660 nm was obtained. An enlarged image of the final output spectrum between 1560 nm and 1660 nm is given in Figure 8c. It can be observed that the flatness of the generated OFC was about 6 dB over the range of 1565–1650 nm. Due to the spectral symmetry on both sides of the central wavelength, the final output spectrum of the OFC at the output of the CASD-TMF had a 6 dB bandwidth of 170 nm.

## 5. Conclusions

In summary, a scheme based on the designed CASD-TMF was proposed for generating broadband OFC. In the modelling of the TMF, we considered a geometry with two layers of regular hexagonal air holes, and the dispersion characteristics of the cross-section were calculated to achieve optimized design parameters. By considering the influence of different nonlinear effects, the ultrashort pulse evolution in the AD zone and ND zone of the CASD-TMF was analyzed based on GNLSE. Then, by injecting a 20 GHz high-repetition-rate seed OFC into the optimized CASD-TMF, the output spectra after passing through different TMF zones were demonstrated, and highly coherent combs with a 6 dB width covering over 170 nm were obtained. The OFC generator based on the optimized CASD-TMF can effectively break through the bandwidth limit of electro-optic modulation, which will be useful for exploring high-repetition-rate, multi-wavelength light sources for various integrated microwave photonics and ultrafast optical signal processing applications.

## Figures and Tables

**Table 1:** Some of the main simulation parameters of ND TMF and AD TMF.

Zone	$\begin{array}{c} D_{2}\\ \left(\mu m\right) \end{array}$	$\begin{array}{c} \beta_{2}\\ \left({\mathrm{ps}}^{2}/\mathrm{km}\right) \end{array}$	$\begin{array}{c} \beta_{3}\\ \left({\mathrm{ps}}^{3}/\mathrm{km}\right) \end{array}$	$\begin{array}{c} A_{eff}\\ \left({\mu m}^{2}\right) \end{array}$	$\begin{array}{c} \gamma \\ \left(m^{-1}W^{-1}\right) \end{array}$	$\begin{array}{c} Loss\\ \left(\mathrm{dB}/m\right) \end{array}$
ND TMF	0.6	9.4601	−0.0085	2.55	2.2255	1.5
AD TMF	0.72	−45.9939	0.0703	2.11	2.6896	1.5
